# Cases Occurring in the Practice of S. W. Ritchey, M. D.

**Published:** 1852-03

**Authors:** 


ARTICLE III.

*Cases occurring in the Practice of* SAMUEL W. RITCHEY, M. D., of Newtown, Indiana.

DR. EVANS:—I have often thought of reporting two or three cases which came under my observation some time since, thinking that perhaps they might be somewhat rare, and consequently interesting to the profession.

The first was a Surgical case. A fleshy boy about twelve year of age, with a basket hanging upon his arm, was shot with a tolerably large rifle ball which had been carried near two hundred yards. It entered his arm near the insertion of the Deltoid muscle, whence it passed almost directly to the bone, but inclining perhaps a little upward. Dr. Crawford (now dead) and myself having been called in the case, concluded to cut down upon the bone in search of the ball, which, as it could not be felt on the opposite side of the arm, and evidently had not passed through it, we concluded would most likely be found near the front where it struck the bone. We thought, as the force of the ball must have been very nearly exhausted, it might have slid a little round or up the bone. But upon cutting down to the bone, we were surprised to find a hole, into which the index finger could readily enter; but the ball had not passed through, and was not in reach of the finger or the probe. This hole was somewhat longer in the direction of the bone. You may not suppose our mortification was so great, as that of a distinguished Surgeon, whom I saw, a few years ago dip twice, and deep, into an enlarged *scrotum* which he declared to be a Hydrocele, and found no water; but we found we were in water, the depth of which we did not understand. Our conclusion here was, that the ball had passed to the centre of the bone, and not having force to pass through, made its way up the bone, favored by its cavity, and lodged a little above the outward opening. Trephining the bone and amputation, even, were thought of, but fortunately neither of these operations were performed. The result would have proven either of them cute, certainly. Some lines of Dr. Watts, are here appropriately suggested to my mind :

> " So when a raging fever burns,
> We change from side to side by turns;
> And it's but a poor relief we gain,
> To change the side, but keep the pain."

The wound was dressed, and in a few weeks entirely healed, and after a few months the ball was discovered under the Integuments close to the inferior Costa, about one to two inches above the inferior angle of the Scapula.

From the above account, which, so far as I can see, is exactly correct, what was the more probable course of the ball in arriving at this point? Did it enter the bone and pass out at a point some distance from its entrance; or could it drive that hole in the bone, rebound, and take its course outside ?

Another case. A Mr. L. was taken with a lingering Typhoid fever in the month of June. He had some cough, and pain in the left side, and over this same side there was great dullness on percussian. After four or five weeks he got up, but the pain and dullness still continued. He visited round for some three or four weeks, (but still feeble,) when a tumor began to show itself immediately above the Clavicle. I questioned first whether it were an Aneurism or an Abscess. I soon satisfied myself, however, that it was the latter. In two or three weeks from its first appearance, I was called to open it. A large quantity of pus was discharged. I then expressed to some of the relatives my fear that he would sink under the waste. I suspected it proceeded from the Lung, and tried to probe to satisfy myself on this point, but did not succeed. He sank down for weeks, emaciated as a man in Phthisis, with a well developed hectic fever. When he died, I opened the breast, and found the left lung entirely destroyed. Nothing like lung in any state was discovered. The passage from this cavety of the thorax to the abscess in the neck was very clear, being nearly an inch in width. The right lung, so far as examined, appeared healthy. Such cases, it appears to me, are incurable, and therefore I ventured to give this report, though in some respects imperfect. This was most likely a case of Tubercular disease. Several of the family have died of Consumption.

